# Indian Hemp—Its Active Principle

**Published:** 1848-08

**Authors:** 


*Indian Hemp; its Active Principle.*—M. De Courtive has presented to the School of Pharmacy, Paris, a thesis on the Cannabis Indica, which attracted considerable attention. His inquiry was directed to it by the effects he noticed it to produce upon the lunatics in the Bicêtre. He has submitted the cannabis to analysis, procuring the plant from Algiers, or from Indian seeds reared in France. The active principle he states to reside in a resin which he extracted by a complicated process of maceration, and the action of alcohol. From nine to ten parts of this resin were procured from 100 parts of the plant, the larger proportion being furnished by the Algerine drug. He wishes to call the resin, cannabina, and states that one grain and two thirds, or even, in some temperaments, half that quantity, produced an equal effect with half a drachm of thick extract.

An alcoholic extract obtained directly from the plant may be advantageously employed, but double the quantity must then be used at a dose, since the active principle is more mixed up with inert matters.

M. Courtive has experimented similarly with the cannabis sativa of Italy, and has thence extracted an active resin, which, however, requires to be given in four or five grain doses, to produce an effect. He considers the difference between the cannabis Indica and the cannabis sativa to be insufficient to distinguish them as different species, and in this he agrees with Dr. Boyle and others.

The resin extracted the author describes as deep greenish-brown; of an aromatic, yet nauseous odour: of a hot, acrid, and enduring taste; soluble in cold ether, alcohol, and volatile oils; insoluble in water and dilute spirit. It would appear to contain some fatty matter and chlorophylle.

The leaves are looked upon as the most active parts of the plant.

Indian hemp, we believe, must assume a much more important place in our list of drugs than it has as yet enjoyed; and if a resin of unvarying strength can be readily procured, it will provide us with a most eligible form of administering the drug.—*Lancet.*

